# Representing physiological processes and their participants with PhysioMaps

**DOI:** 10.1186/2041-1480-4-S1-S2

**Published:** 2013-04-15

**Authors:** Daniel L Cook, Maxwell L Neal, Robert Hoehndorf, Georgios V Gkoutos, John H Gennari

**Affiliations:** 1 Biomedical & Health Informatics, Univ. of Washington, USA; 2 Bioengineering, Univ. of Washington, USA; 3 Dep’t of Physiology, Development & Neuroscience, Univ. of Cambridge, UK; 4 Computer Science Dept., Univ. of Aberystwyth, UK

## Abstract

**Background:**

As the number and size of biological knowledge resources for physiology grows, researchers need improved tools for searching and integrating knowledge and physiological models. Unfortunately, current resources—databases, simulation models, and knowledge bases, for example—are only occasionally and idiosyncratically explicit about the semantics of the biological entities and processes that they describe.

**Results:**

We present a formal approach, based on the semantics of biophysics as represented in the Ontology of Physics for Biology, that divides physiological knowledge into three partitions: structural knowledge, process knowledge and biophysical knowledge. We then computationally integrate these partitions across multiple structural and biophysical domains as computable ontologies by which such knowledge can be archived, reused, and displayed. Our key result is the semi-automatic parsing of biosimulation model code into PhysioMaps that can be displayed and interrogated for qualitative responses to hypothetical perturbations.

**Conclusions:**

Strong, explicit semantics of biophysics can provide a formal, computational basis for integrating physiological knowledge in a manner that supports visualization of the physiological content of biosimulation models across spatial scales and biophysical domains.

## Background

Researchers developing large scale, integrative projects such as the Physiome[1], the Virtual Physiological Human (http://www.vph-noe.eu), and the Virtual Physiological Rat[2] have aimed to use biomedical ontologies to improve access to biomedical knowledge resources and to analyze and even integrate some of their content. As contributors to some of these projects, we have aimed to create computable knowledge networks of biological processes and their participants, which we term a “PhysioMap”. These PhysioMaps represent and explain physiological hypotheses that are embodied in biosimulation models, and are designed to aid in information retrieval and model integration across biomedical disciplines and knowledge resources. PhysioMaps are formalized versions of the kinds of informal diagrams that are routinely used in papers and presentations for representing the physiological content of datasets, models, and research domains. Examples of PhysioMaps are the reaction pathway diagrams as generated by KEGG[3], Reactome[4], and the BioModels[5] resources. See for example, the SBML layout package, sbmllayout.sourceforge.net, for visualizations of SBML models. Similarly, for Reactome, the BioPAX formalism[6] provides the basis for a node-and-arc representation of processes. In all of these, nodes represent portions of chemicals that are linked by arcs that represent reaction pathways. We argue that this node-arc-node representation of physiological processes generalizes across temporal and structural scales as well as biophysical domains such that it is applicable to chemical diffusion, heat flow, transmembrane ion currents, as well as more familiar domains such as fluid flow.

**Figure 1:**
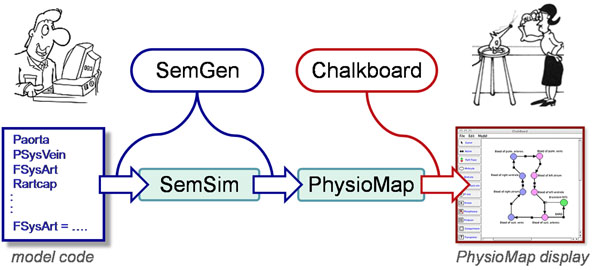
Workflow by which mathematical modelers can derive PhysioMaps, using SemGen software, for experimentalists to display and query in Chalkboard software.

Projects that aim to integrate biomedical knowledge across multiple scales would benefit from holistic PhysioMaps that represent the known (or hypothesized) connections between, say, the expression of a gene, its impacts on cell signaling, and ultimately its effects on macroscopic physiology and pathology. Current resources, such as the Gene Ontology, are useful for annotating data and models in terms of defined biological process classes, yet their underlying knowledge architecture lacks formal relations for linking processes to their physical participants or for representing causal chains of processes. Our goal has been to generalize such mappings to all structural/temporal scales and to other biophysical domains to serve as valuable knowledge resources for displaying the scope of integrative projects in biomedicine (as in the physiome, VPH, VPR) and as a channel for communication between mathematical biophysicists, who express their ideas in computer code, and nonmathematical experimentalists who express their ideas diagrammatically and qualitatively (as illustrated in Figure 1).

**Figure 2:**
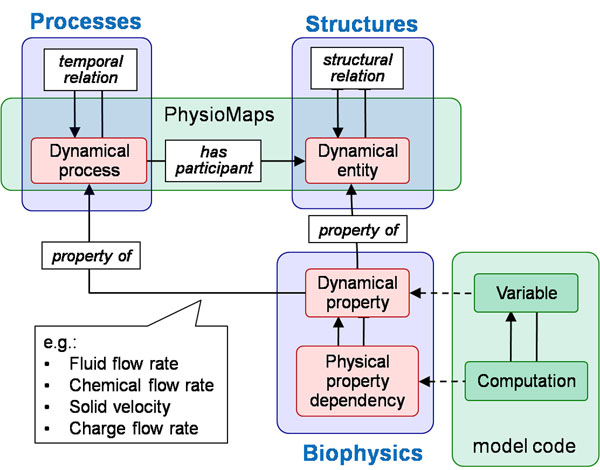
Our knowledge architecture illustrates how PhysioMaps relate to a tripartite structure for physiology (processes, structural entities, and biophysical dependencies) that is based on the OPB.

With these goals, we have developed a software workflow (Figure 1) and a knowledge architecture (Figure 2, next section)by which modelers using SemGen software[7]can read and parse biosimulation model code (in SBML, JSim, or CellML) into a SemSim model from which a PhysioMap file is extracted. Finally, our Chalkboard software[8] can import this PhysioMap file for display as well as qualitative, cause-effect exploration. Our approach begins with the physics-based physiological knowledge already formally expressed as biosimulation code by physiologists and bioengineers.

### Biosimulation models as formalized physiological knowledge

Biosimulation models are developed and curated to formally express physiological hypotheses about how complex biological systems work and to quantitatively test these hypotheses against experimental data. Thus, for generations, physiologists and biophysicists have encoded and archived models in a variety of computational languages including SBML (sbml.org), CellML (http://www.cellml.org), and JSim’s MML(http://physiome.org/jsim/), as well as more general-purpose languages that are not designed for the biomedical domain (e.g., MATLAB, Fortran, C++, etc.). Such biosimulation models represent formal, physics-based expressions of one or more key biomedical hypotheses that are of such interest as to warrant the difficult, time-consuming, and error-prone effort required to encode, debug, and evaluate the model. By focusing first on such physics-based simulation models, we expect to stress-test our approach against formally correct biophysics to assure ourselves that our methods are, in fact, sufficiently rigorous to be applied to any physical representation of physiological processes.

## Results

In the following we describe our logical schema and computational architecture for PhysioMaps, describe our methods for creating PhysioMaps from biosimulation model code, and then present initial visualizations of example PhysioMaps derived from models.

### PhysioMap knowledge architecture

PhysioMaps and SemSim models are based on a foundation provided by the Ontology of Physics for Biology (OPB)[9]. The OPB represents entities and relations used in engineering systems dynamics[10,11] and biological network thermodynamics[12]. It thus leverages formal analogies between physical properties and their quantitative dependencies to span multiple structural and temporal scales and across multiple biophysical domains including chemical reactions, fluid flow, diffusion, electrophysiology, etc. For example, the dependency of chemical reaction rates on reactant concentrations is analogous to the dependency of fluid flow rates on fluid pressures (or ion currents on electrochemical gradients, and so forth).As in many upper-level ontologies (e.g., the Basic Formal Ontology, http://ifomis.org/bfo/) OPB physical entities are continuants that participate in processes (“occurrents”). Participant types can be annotated as classes in various biomedical ontologies such as FMA[13], CL[14]), GO[15], and ChEBI[16]. The OPB is orthogonal to other ontologies because entities and processes are defined dynamically in terms of thermodynamic quantities so that OPB:*Dynamical entity* is defined as “the bearer of a portion of thermodynamic energy” and OPB:*Dynamical process* as “the flow of thermodynamic energy between participating dynamical entities”.

Figure 2 illustrates the tripartite representational schema by which PhysioMaps are based on the OPB representational architecture: (a) **dynamical entities** and their structural relations (e.g., parthood), (b) **dynamical processes** and their temporal relations(e.g., temporal parthood), and (c) the **biophysical dependencies** that constrain how the properties of entities change over time and govern the time-course of processes as they occur in time. These dependencies may be definitions such as Newton’s law or constitutive laws such as Ohm’s law that describe empirically observed dependencies between forces and flows. Next, we describe each of these three classes in more detail.

Dynamical entity classes (OPB:*Dynamical entity*) formally represent the physical entities (e.g., organs, cells, molecules) that participate in biological processes in the domains of anatomy, biochemistry and physiology. Structural relations among these physical entities include “part-of” and “connected-to” [17] whereby, for example, a cardiomyocyte is *part-of* wall of left ventricle that is *connected-to* wall of right ventricle. Process classes (OPB:*Dynamical process*) represent occurrences whereby physical entities undergo changes of composition (e.g., losing a part) or magnitudes of a physical property (e.g., size or material flow rate). Processes are temporally related by relations such as *precedes* and *has-process-part* that are analogous to structural relations. Property classes (OPB:*Dynamical property*) represent physical observable attributes of dynamical entities and processes by which the physical state of an entity and the progress of a process is observed to occur. OPB:*Physical property dependencies* represent the quantitative relations by which the values of physical properties depend upon one another according to the definitions and laws of physics. Ohm’s law, for example, is encoded as OPB:*Electrical resistive flow dependency* which is defined to be a relation between the difference between two voltages and the flow of electrical current. OPB:*Fluid resistive flow dependency* is an analogous dependency for fluid pressures and flows. In each case, such dependencies relate the rate of a physical process to the physical states of their participating physical entities where, for example, the rate of a blood flow process depends on the fluid pressure in the source blood pool and on the pressure in the sink blood pool. It is precisely these dependency relations by which our software identifies participants and processes by parsing the annotated mathematical code of simulation models.

Building from this foundational OPB schema, we work with three computational artifacts used in the workflow of Figure 1: (1) PhysioMaps, (2) model code, and (3) SemSim models. PhysioMaps are files, currently encoded in XML, that consist of a set of dynamical processes linked by the set of dynamical entities that are its participants. Model code is the code-level implementation of biosimulation models as written in any of the various modeling languages available (including SBML, JSim, and CellML). SemSim (semantic simulation) models are OWL-encoded ontologies that map model variables and equations to OPB property and dependency classes (see rightmost part of Figure 2) while retaining the model equations and parameter values and a link to the model file itself.

As developed above, a SemSim model makes explicit the biophysical knowledge implicit in biosimulation model code. SemSim models combine biophysical and structural views of particular biosimulation models in which each model variable and parameter is annotated using a composite annotation[18,19] against a defined subset of orthogonal biomedical ontologies (we have primarily used OPB, ChEBI, and the FMA). Furthermore, SemSim creates a map of the mathematical dependencies between variables so that, for example, a chemical reaction rate variable will depend on a concentration variable for each reactant and on one or more rate-law parameters.

### Creating PhysioMaps from model code

At this stage of PhysioMap development, we aim to represent only two kinds of physical process. First, we consider physical flows (subclasses of OPB:*Energy flow process*) during which a quantity of “stuff” (e.g., blood, molecules; attended by a corresponding amount of energy) flows from one physical entity to another (e.g., blood flow from aorta to femoral artery). Second, we consider OPB:*Modulation processes* during which the value of one physical property (of an entity or process) directly affects the value of some other physical property without significant energy flow. For example, in pathway databases (e.g., Reactome, KEGG) enzyme reaction process rates are modulated by the concentrations of activating or inhibiting molecules without regard to the details of the actual reaction kinetics.

**Figure 3:**
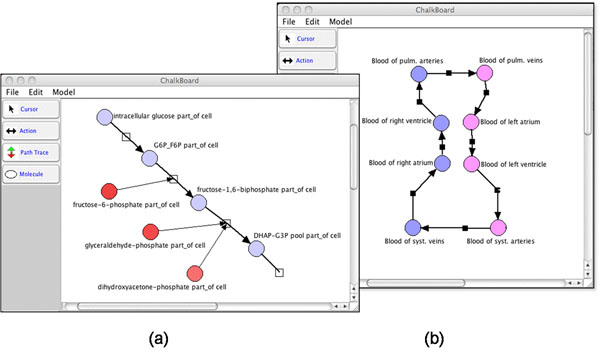
Prototype PhysioMaps as visualized by the Chalkboard system (Cook et al., 2007): (a) shows a simple model of glycolysis and (b) shows blood flow processes in a cardiovascular (CV) model.

For flow processes, we provide two examples (see Figure 3) to demonstrate how we generate PhysioMaps from biosimulation models. For macroscopic flow processes, consider a model of cardiovascular dynamics. First, we map model variables to physical property classes (e.g., OPB:*Fluid pressure*) that are linked via *property-of* relations to the physical entity (e.g. FMA:*Blood in aorta*) that bears the property. For example, a flow-rate variable (e.g., “F_LV-aorta_”) representing the flow of blood from one FMA:*Portion of blood* (e.g., in left ventricle) to another (e.g., in the aorta) is annotated as an individual of class OPB:*Fluid flow rate*. Such a variable is, identically, an attribute of the flow-source entity (e.g., FMA:*Blood in left ventricle*), the flow-sink entity (e.g., FMA:*Blood in aorta*) and the flow process itself (e.g., OPB:*Fluid flow process*). Such flow variables are annotated as subclasses of OPB:*Dynamical flow rate* such as OPB:*Fluid flow rate* (e.g., for blood or air flows) or OPB:*Chemical flow rate* (e.g., for molar chemical flows) that are attributes of a OPB:*Fluid flow process*or of a OPB:*Chemical flow process*, respectively. In subsequent work we plan to generalize our multiscale and multidomain approach to include OPB:*Physical process* classes such as OPB:*Fluid capacitive process* and OPB:*Transducer process* by which thermodynamic energy is stored and redistributed amongst process participants. For each such flow property, SemGen infers and instantiates its corresponding flow process as a PhysioMap element.

For molecular flow processes, consider a models of glycolysis. For example, Figure 3a shows a fragment of the glycolysis pathway derived from a curated, annotated SBML model from the BioModels database (model ID: BIOMD0000000236). In some pathway models, such as this one, reactants, products, and reaction modifiers are explicitly tagged, and thus, SemGen can automatically generate a PhysioMap from the tags. In cases where such explicit semantic tags are unavailable, SemGen derives a computational dependency network by parsing the SBML-encoded equations that include flow process variables (i.e., those annotated as OPB:*Dynamical flow rate* subclasses). SemGen then: (1) identifies physical property variables upon which the flow variable depends and which are annotated to be of the same biophysical domain as the flow variable (e.g., OPB:*Fluid kinetic domain*) and (2), identifies force (OPB:*Force property*) or amount (OPB:*Amount property*) variables that can play the role of a driver for the flow. From such relations, SemGen infers that the entities that bear the force or amount properties are thus participants in the process. SemGen then identifies downstream process participants by finding force and amount variables that are mathematically dependent on the flow term.

### Displaying and interrogating PhysioMaps

Having created a SemSim model, we derive and export a PhysioMaps from SemGen into Chalkboard for display and cause/effect querying. Chalkboard is an editor for the BioD biological description language[20] that is similar in design intent to SBGN Process Description language [21], SBML Layout Tools (http://sbmllayout.sourceforge.net/SBMLLayout/Welcome.html), and ChiBE for BioPAX models[22]. In our prototype demonstrations, we have extended Chalkboard to read PhysioMap files and to represent processes as rectangles with arrows that link to circle icons that represent participating entities. Figure 3a shows, on the left side, a PhysioMap representation of a portion of glycolysis, derived from the BioModels model BIOMD0000000236, and on the right side, a portion of a cardiovascular model derived from a lumped-parameter model of cardiovascular dynamics [23]. In Figure 3a, the arrows represent molar chemical flows (except for the modulatory processes), whereas in Figure 3b, the arrows are fluid flows between portions of blood located in a circuit of blood vessels and heart chambers.

PhysioMaps in Chalkboard can be interrogated about qualitative perturbations introduced anywhere in the network. Chalkboard’s Path Tracing feature supports “thought experiments” to display the consequences of researcher’s interpretations of how a dynamic system might behave for a given set of hypothetical experimental conditions. Thus, experimental perturbations (e.g., an increment in intracellular glucose in Figure 3a, or in the amount of aortic blood in Figure 3b) can be propagated through a functional network as increments and decrements in the amounts or flow-rates of connected participants and processes. To do this effectively, all modulators (e.g. enzymes in biochemical reactions) must be annotated with a polarity—are the modulators inhibitors or stimulators? As implemented in Chalkboard, Path Tracing can trace “A-to-B” pathways in complex networks, detect positive- and negative-feedback loops, and display the qualitative (up or down) responses of all affected processes and participants in the network.

## Discussion and conclusions

We have developed the idea of PhysioMaps and some prototype implementations that extend our current SemSim technology to make explicit the connections among biological processes, the physical entities that participate in those processes, and the biophysics that determine how biological processes occur over time. This tripartite formal view of physiology (as in Figure 2) will better enable knowledge and model integration across resources, which in turn will enable improved understanding and better models of physiological processes.

Our approach is domain- and scale-independent. Prior process visualization efforts such as for BioPAX or SBML models apply only to a single biophysical domain—mass action chemical kinetics —at a single scale—subcellular biochemical reactions. In contrast, because our approach is based on foundational theories of systems dynamics and classical physics, PhysioMaps generalize across all biophysical process domains (e.g., fluid flow, diffusion, or electrophysiology) as required for integrated analysis of multiscale physiological systems.

### PhysioMap next steps

We are aware of a number of limitations of our work to date, and these help direct the next steps in our research. First, our current Chalkboard implementation does not leverage modern graph layout algorithms and drawing packages; although these user interface details do not affect our theory and approach to modeling, they certain do affect the ability of outside users to test or use our software.

Second, although we have generated a number of example PhysioMap files, we have not yet carried out any sort of exhaustive survey of biosimulation models to understand where are methods work well and where they do not. We expect that the generation of PhysioMaps can never be a fully automatic process for all models. As should be clear from Figure 2, a PhysioMap is an abstraction, both of the SemSim model, and even more so, of the underlying biosimulation model. Thus, choosing what to abstract and what to retain will always require some human guidance. However, our expectation is that we can nearly automate the process for certain classes of biosimulation models, by leveraging common assumptions and characteristics of those models (e.g., the class of all biochemical reaction models that are encoded in SBML).

Third, a challenge we face is that our approach depends on understanding the sources, sinks and mediators of specific flows in a model. Where models include explicit annotations to specify these (as in many models in the BioModels repository), we can generate PhysioMaps in a fully automatic manner. However, lacking these annotations, our approach depends on parsing and deriving these semantics based on the potentially complex mathematical dependencies among flow process variables and the forces and amounts of participating physical entities. Such dependencies can obscured by intervening variables and equations that may not have clear semantics. Future versions of our SemGen tool will make it possible to create and edit such links between processes and their participants to ensure that PhysioMaps generated from SemSim models accurately represent the physiological architecture of the model.

The ability to visualize and perturb biosimulation models (via thought-experiments) is a significant step toward demystifying biosimulation modeling results, and can potentially improve how biosimulation is used to direct experimental research. However, to develop and refine their hypotheses, experimenters will need to be able to dynamically combine and modify PhysioMaps. If a researcher integrates multiple PhysioMaps that represent the same biophysical process (with the same participants and properties), then this process should be uniquely represented in the merged system. Our prior work in merging SemSim models may be applicable to this challenge since integrating SemSim models requires identifying and resolving the components of the models that are semantically identical. In the long run, for large, integrative projects to succeed (e.g., the Virtual Physiological Human or the Virtual Physiological Rat[2]), this sort of PhysioMap merging and visualization capability is essential.

Our vision is to develop multiscale/multidomain physiological pathway maps, patterned after biochemical pathway maps, that represent the physiological content of biological models and datasets for use in large-scale physiological integration projects. These PhysioMaps should help researchers (a) understand the physiology implicit in the mathematics of biosimulation models, (b) manipulate and perturb those models in a graphical manner, and (c) combine and modify PhysioMaps to develop new experimental ideas or hypotheses that will drive research forward toward a more comprehensive understanding of biological processes.

## Competing interests

The authors have no competing interests in this work.

## Authors' contributions

All coauthors participated in numerous discussions leading to the development of these ideas. MLN led all of the development work with SemGen and SemSim models, including the annotation and development of the example models. DLC led the writing of this manuscript, as well as all Chalkboard and OPB development work. JHG and MLN contributed to the writing and editing of the manuscript.

## Declarations

This supplement was partially funded by the VPH Network of excellence, EC FP7, project #248502.

This article has been published as part of *Journal of Biomedical Semantics* Volume 4 Supplement 1, 2013: Proceedings of the Bio-Ontologies Special Interest Group 2012. The full contents of the supplement are available online at http://www.jbiomedsem.com/supplements/4/S1
